# A new esophageal gland transcriptome reveals signatures of large scale de novo effector birth in the root lesion nematode *Pratylenchus penetrans*

**DOI:** 10.1186/s12864-020-07146-0

**Published:** 2020-10-23

**Authors:** Paulo Vieira, Jonathan Shao, Paramasivan Vijayapalani, Thomas R. Maier, Clement Pellegrin, Sebastian Eves-van den Akker, Thomas J. Baum, Lev G. Nemchinov

**Affiliations:** 1 USDA-ARS, Molecular Plant Pathology Laboratory, Beltsville, MD 20705-2350 USA; 2 School of Plant and Environmental Science, Virginia Tech, Blacksburg, VA 24061 USA; 3 Department of Plant Pathology and Microbiology, Iowa State University, Ames, IA 50011 USA; 4 Department of Plant Sciences, University of Cambridge, Cambridge, UK

**Keywords:** Root lesion nematodes, Host-pathogen interaction, Secretome, Esophageal gland cells, Pioneer effectors

## Abstract

**Background:**

The root lesion nematode *Pratylenchus penetrans* is a migratory plant-parasitic nematode responsible for economically important losses in a wide number of crops. Despite the importance of *P. penetrans*, the molecular mechanisms employed by this nematode to promote virulence remain largely unknown.

**Results:**

Here we generated a new and comprehensive esophageal glands-specific transcriptome library for *P. penetrans.* In-depth analysis of this transcriptome enabled a robust identification of a catalogue of 30 new candidate effector genes, which were experimentally validated in the esophageal glands by in situ hybridization. We further validated the expression of a multifaceted network of candidate effectors during the interaction with different plants. To advance our understanding of the “effectorome” of *P. penetrans*, we adopted a phylogenetic approach and compared the expanded effector repertoire of *P. penetrans* to the genome/transcriptome of other nematode species with similar or contrasting parasitism strategies. Our data allowed us to infer plausible evolutionary histories that shaped the effector repertoire of *P. penetrans*, as well as other close and distant plant-parasitic nematodes. Two remarkable trends were apparent: 1) large scale effector birth in the Pratylenchidae in general and *P. penetrans* in particular, and 2) large scale effector death in sedentary (endo) plant-parasitic nematodes.

**Conclusions:**

Our study doubles the number of validated *Pratylenchus penetrans* effectors reported in the literature. The dramatic effector gene gain in *P. penetrans* could be related to the remarkable ability of this nematode to parasitize a large number of plants. Our data provide valuable insights into nematode parasitism and contribute towards basic understating of the adaptation of *P. penetrans* and other root lesion nematodes to specific host plants.

**Supplementary information:**

**Supplementary information** accompanies this paper at 10.1186/s12864-020-07146-0.

## Background

The genus *Pratylenchus* (Pratylenchidae) comprises more than 100 valid species, with some of them being highly important due to the damage they cause in economically important crops [1, 2]. Members of the *Pratylenchus* genus are known as root lesion nematodes (RLNs) and belong to the third most important group of plant-parasitic nematodes (PPNs) [3]. One of the common features of these migratory nematodes is their dynamic behavior within the host roots, i.e. nematodes do not become sedentary and are able to migrate in and out of the roots, causing extensive damage as they do so. In the absence of a host crop, most of these polyphagous nematodes are able to survive on weeds or adapt their survival mechanisms (e.g. anhydrobiosis), which make them difficult to control [4].

*Pratylenchus penetrans* is one of the most successful and economically devastating RLN species with a wide range distribution, associated with more than 400 hosts worldwide [4]. The infection process of *P. penetrans* in different plants has been comprehensively investigated (e.g. [5, 6]). Like other RLNs, it can enter the plant along the entire length of the root. Once inside of the roots, nematodes migrate and feed almost exclusively from the cortical cells, where they cause mechanical damage, browning, and necrosis of the root tissue. Infection of the roots by *P. penetrans* often results in the release of phenolic compounds, oxidation of which has been associated with the browning of the root tissues [4]. Complex networks of defense genes and secondary metabolites have been identified in alfalfa as part of host responses to *P. penetrans* infection [7]. Nevertheless, the molecular mechanisms that RLNs employ to promote virulence remain largely unknown.

An important feature of all PPNs is the presence of a repertoire of secreted proteins (known as effectors), which are critical components determining the outcome of the plant-nematode interactions. The majority of these effectors are synthesized in the esophageal glands of PPNs (one dorsal and two sub-ventral glands) and are ultimately secreted through the nematode stylet into plant tissues [8]. During infection, PPNs can deploy dozens of different effectors that are capable of manipulating and suppressing key molecular pathways of the plant in order to complete their life cycle. Although a growing number of nematode effectors have been analyzed [9], different parasitism strategies, host range, variability, and composition of the effector repertoire complicate their identification and characterization.

Over the past decade, a rapid advance of genomic and transcriptomic sequencing approaches has greatly accelerated the identification of nematode effectors. Specialized techniques for RNA extraction from single cells have been adapted for the esophageal glands of PPNs, which resulted in generation of gland-specific libraries for different nematodes and identification of a significant number of candidate effector genes [10].

The explanation for the ability of *P. penetrans* to parasitize a wide range of hosts has yet to be determined, but it may, at least in part, lie in its effector repertoire. The annotation of PPN effector proteins often relies on sequence similarity to known effectors, or prediction via promoter motif elements (e.g., [11, 12]). Sequence data from both sedentary and migratory plant-parasitic nematodes have provided insights into the conservation of some effectors, which presumably reveals basic function(s) required for nematode parasitism [9]. Contrary to sedentary PPNs, RLNs do not induce complex feeding sites like syncytia or giant-cells and their effector repertoires are likely to reflect this difference. In our previous work, we have generated an extensive catalogue of candidate secreted proteins for *P. penetrans* [13]. A significant proportion of those predicted proteins have no homologues, and their functions are unknown, indicating a distinct complement of the *P. penetrans “*effectorome”. More recently, we determined the spatial expression of 23 candidate effectors within the esophageal glands of *P. penetrans* by in situ hybridization assays [14, 15]. These proteins comprised common signatures of PPN effectors with a diversity of known functions (e.g. cell wall-degrading enzymes, CWDEs). In addition, a set of pioneer genes specific to *P. penetrans,* that possesses unique features has been identified [14]. A high proportion of the predicted secreted proteins of *P. penetrans* showed no similarities with proteins deposited in public datasets, which raises the possibility that additional effectors for this species remained unexplored.

Here, we generated a new and more comprehensive esophageal glands-specific transcriptome library of *P. penetrans.* An in-depth analysis of this transcriptome led to the identification of new candidate effector genes for this species, which are specifically expressed in the esophageal glands. Many of these novel genes are phylogenetically restricted to *P. penetrans* or the *Pratylenchus* genus.

## Results

### A more complete roster of candidate effector genes of *Pratylenchus penetrans*

The current library generated from the esophageal glands of *P. penetrans* resulted in ~ 150 million raw reads. The raw reads were cleaned and approximately 99 million high-quality reads were mapped against the 23,715 transcripts previously generated from the same *P. penetrans* isolate [13], and against the genomic sequence of the recently discovered root lesion nematode virus 1, which was found to be associated with the same nematode isolate [16]. As it is common in similar datasets [12], a large proportion of the reads (~ 90 million) mapped to the nematode *18S* and *28S* ribosomal genes. Surprisingly, approximately two million reads mapped to the genome of root lesion nematode virus 1, thus confirming our earlier observations on the virus localization adjacent to the nematode esophageal glands [16]. The remaining 6,380,993 reads mapped to 11,514 nematode transcripts, with coverage ranging from 1 to 239,619 reads. A tenfold increase in the transcripts number observed in the present dataset as compared to the previous gland cell sequencing attempts of *P. penetrans* ([10]; *n* = 1098 versus *n* = 11,514), was largely due to the improved extraction procedures of a technically difficult experiment and higher sequencing depth.

**Fig. 1:**
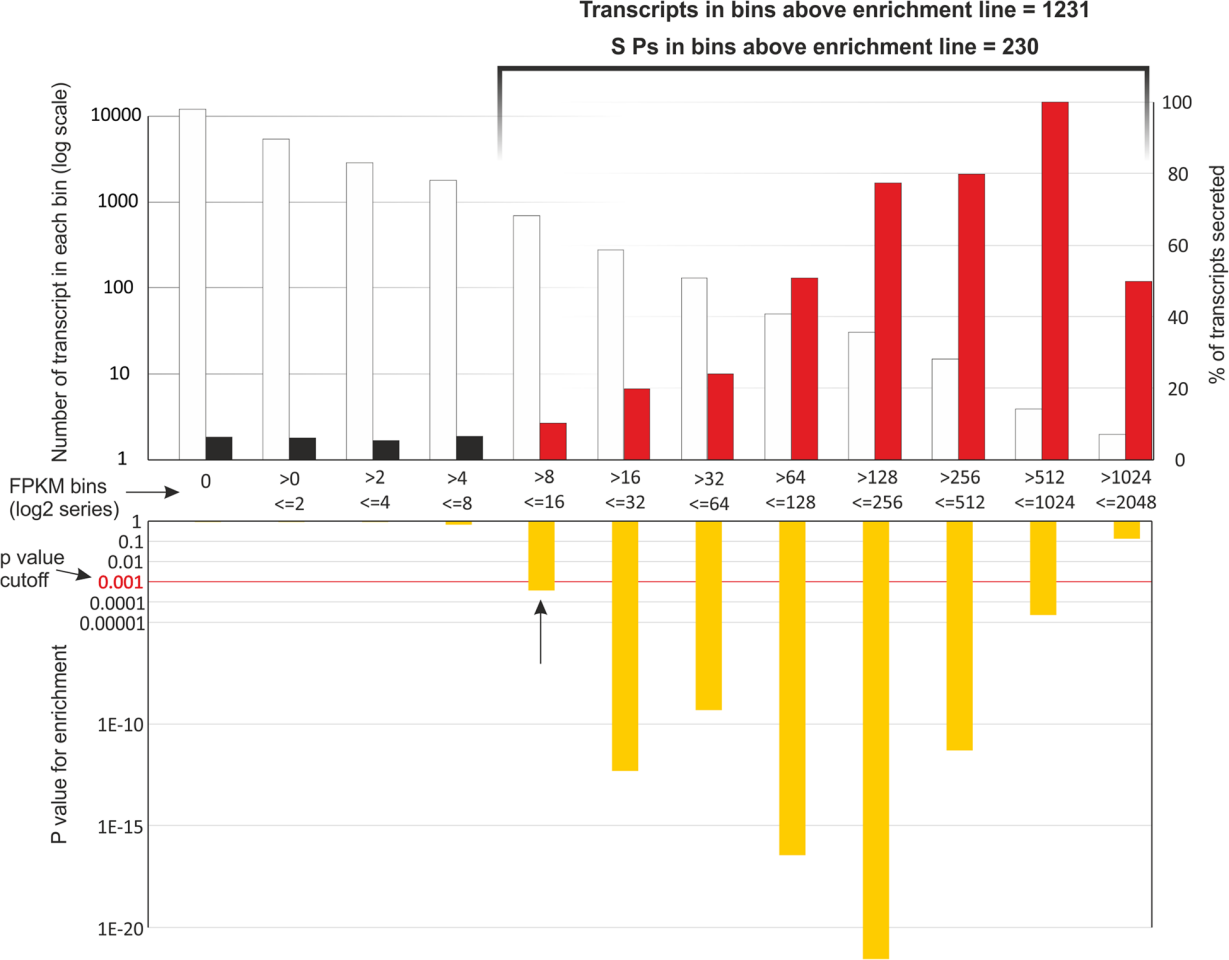
Schematic representation of all gland-cell transcripts. White bars were assigned to one of twelve bins (increasing in a Log_2_ series) based on their abundance using the fragment per kilobase of transcript per million mapped reads (FPKM) values in the gland cell library. The proportion of transcripts encoding proteins with a predicted signal peptide (red bars) was significantly enriched (*p-*value between 0.001 and 1e^− 20^, yellow bars) in all bins above FPKM > 8, with the exception of the most highly represented bin due to a low number of transcripts (FPKM > 1024, *p* > 0.1)

Some of the key features for the identification of candidate effectors, which are expected to be secreted by the classical ER-Golgi secretory pathway, are the presence of a signal peptide and absence of a transmembrane domain. Of the 11,514 transcripts with at least one read in the gland cell library, 864 (7.5%) encode putatively secreted proteins. All transcripts were assigned to one of twelve bins (increasing in a Log_2_ series) based on how highly they were represented in the gland cell library (Fig. 1). As representation in the gland cell library increased, two general trends were observed: 1) the total number of transcripts decreased; and 2) the relative proportion of transcripts that encode proteins with a signal peptide increased. The proportion of transcripts encoding proteins with a predicted signal peptide was significantly enriched (hypergeometric test, *p-*value between 0.001 and 1e^− 20^) in all bins above FPKM > 8 (Fragments Per Kilobase of Transcript per Million mapped reads), with the exception of the most highly represented bin due to a low n (FPKM > 1024, *p* > 0.1). In some of the most highly represented bins, 80–100% of transcripts encode putatively secreted proteins (Fig. 1). Assuming representation in the library is a function of expression in the gland cells, these observations support the important secretory function of the nematode esophageal glands. Since transcripts with low coverage in the gland cell library could not be distinguished from those derived from non-specific sequenced RNA originated from tissues potentially adjacent to gland cells (due to a technically challenging gland cell extraction procedure), a highly stringent cut-off value was used as a conservative measure of bona fide gland cell expression to identify transcripts to be further studied in this work. We used the statistically significant enrichment of secretory proteins (*p* < 0.001) as a proxy to establish the minimum coverage bin for further analyses. A total of 230 transcripts from the highly enriched bins (FPKM > 8) encode putatively secreted proteins with no transmembrane domain (Additional file 1: Table S1).

### Mining for new candidate effector genes

**Fig. 2:**
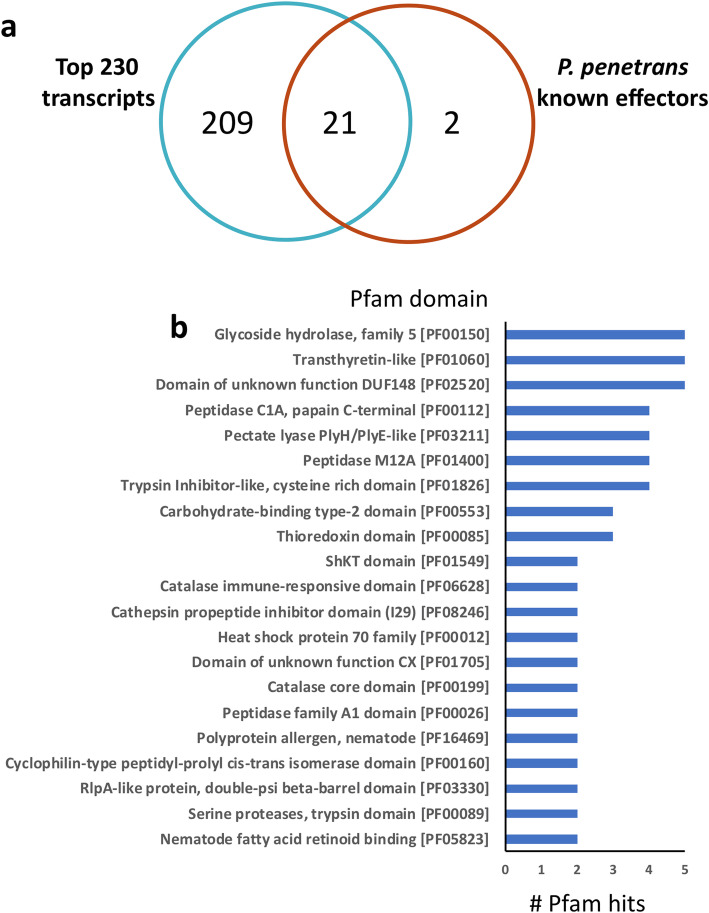
Characterization of the most abundant transcripts encoding putative secreted proteins collected from the esophageal gland library of *Pratylenchus penetrans*. **a** Venn diagram showing the number of experimentally validated candidate effectors within the top 230 most abundant transcripts. **b** Most abundant Pfam protein domains represented within the list of 230 transcripts encoding putative secreted proteins (e-value <1e^− 5^)

To evaluate the validity of this strategy, we first queried for the presence of genes that were previously confirmed in the esophageal glands of *P. penetrans*. Remarkably, 91% (21/23) of the genes validated previously were in this set of 230 transcripts with FPKM > 8 in the gland-cell library (Fig. 2a and Additional file 1: Table S1). These included transcripts encoding proteins with known annotation (e.g. CWDEs), as well as seven previously identified “pioneer effectors” [14]. Most of the 21 previously validated genes ranked among the top of this list, when ranked by representation in the gland cell library (Additional file 1: Table S1). Two previously validated gland cell expressed genes were absent from this set of 230 transcripts: a fatty acid- and retinoid-binding protein (FPKM = 1.21) and a pectin methylesterase (FPKM = 1.50) (Additional file 2: Table S2).

A detailed examination of the remaining 209 transcripts revealed an extensive overlap with genes previously reported to be involved in parasitism of other PPNs (Fig. 2b and Additional file 1: Table S1). For instance, transcripts encoding various proteases [17], transthyretin-like proteins [12, 18], protein disulfide isomerases [19], protease inhibitors [14], an acid phosphatase [20], and a saposin precursor [21] were identified. In addition, some transcripts were similar to putative effectors recently identified for the cyst nematode *Heterodera avenae* (BLAST e-value < 10^− 5^, [22]). It is noteworthy that the majority (133/230, 57%) of the 230 transcripts encode novel proteins lacking any functional annotations or similar sequences in the NR database (BLAST e-value < 10^− 5^).

Given that other candidate effectors could be present below the established cut-off (FPKM < 8), we assessed the distribution of additional known effector genes among those transcripts encoding predicted secreted proteins (Additional file 2: Table S2). This led to the identification of other relevant candidate effectors (1 < FPKM < 8), such as transcripts encoding an esophageal gland protein of the root-knot nematode (FPKM = 7.92), a chorismate mutase (FPKM = 6.7), among other candidate effectors. Taken together, these results suggested that a convincing number of previously identified effectors were highly abundant in the gland-cell library and above the stringent threshold established herein.

### Identification of new candidate effector genes for *Pratylenchus penetrans*

**Fig. 3:**
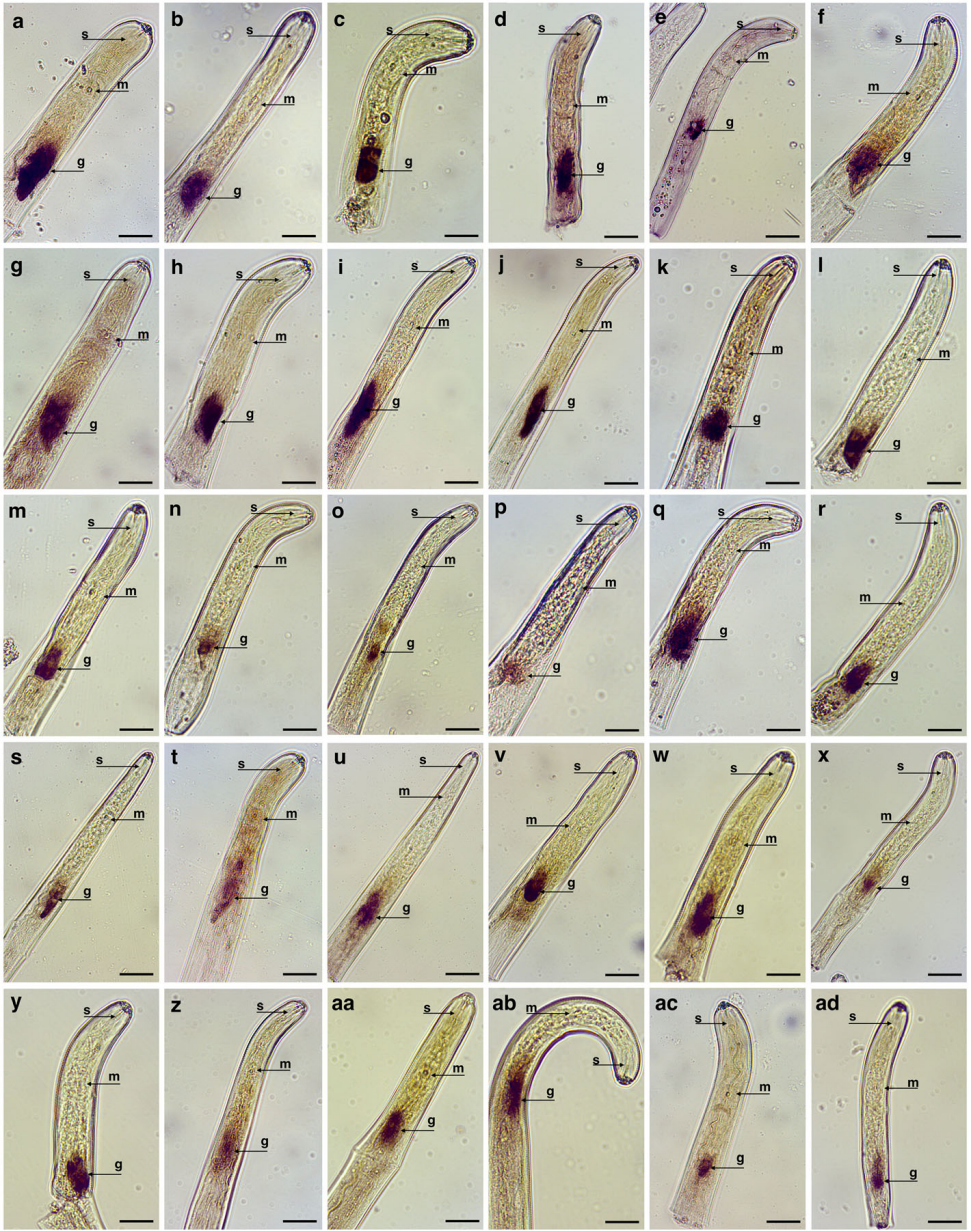
Detection of gene transcripts encoding putative secreted proteins by in situ hybridization. Transcripts encoding thirty different genes were localized in the nematode esophageal glands of *Pratylenchus penetrans* using the corresponding anti-sense DIG-labeled probes. Due to the high variability of the esophageal gland size among different specimens and nematode stages, both dorsal and subventral glands were labelled as esophageal glands. Details regarding each gene annotation and description are presented in the same order as sorted in Table 1. g: esophageal glands; m: median bulb; s: stylet. Bars = 20 μm

From the list of 230 transcripts, 68 candidates were selected for an in-depth analysis based on the mining results (Additional file 1: Table S1). The selected transcripts included those encoding 52 novel proteins (i.e., those with no annotation) and 16 genes with predicted functional annotation. Out of the 68 candidates tested, 30 transcripts were specifically localized in the nematode esophageal glands, as demonstrated by in situ hybridization assays with the corresponding anti-sense probes (Fig. 3). Hybridization with control probes of sense orientation revealed no signal (Additional file 3: Fig. S1). No signal (not to be confused with a conflicting signal) was detected in the nematode sections for the remaining genes, which suggests that additional candidate effectors could be added to this list following probe optimization.

**Fig. 4:**
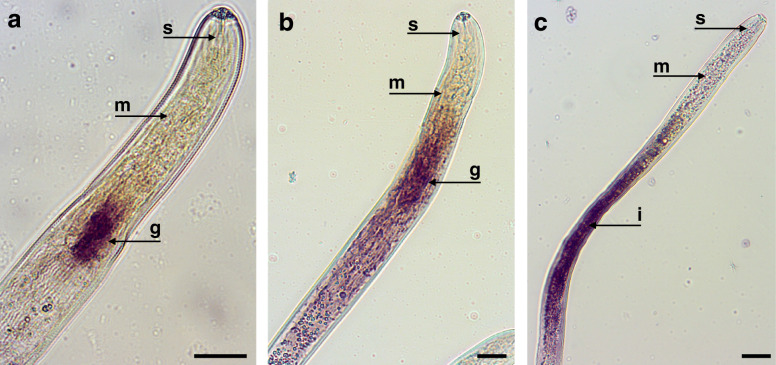
Detection of gene transcripts encoding putative proteins without signal peptide by in situ hybridization. (**a**) ShK domain-like protein, and (b-c) 14–3-3 protein. g: esophageal glands; i: intestine region; m: median bulb; s: stylet. Bars = 20 μm

To determine whether transcripts above the FPKM threshold that encode proteins without a predicted signal peptide were also specific to the esophageal glands, we performed in situ hybridization for the following four transcripts: a transcript encoding an ShK domain-like protein (FPKM = 213.3), a translationally-controlled tumor protein (TCTP, FPKM = 92.4), one novel protein (FPKM = 50.4) and a 14–3-3 protein (FPKM = 25.3). A positive labeling localized in the esophageal glands area was found for transcripts encoding the ShK domain-containing protein (Fig. 4a). Although the signal associated with transcripts encoding the 14–3-3 protein (Fig. 4b) was detectable in the glands, it was also observed along the intestine region of some nematodes (Fig. 4c). No signal was observed for the two remaining genes, which was also true for all control probes of sense orientation (Additional file 4: Fig. S2).

Following these results, we also validated the presence of the CAA [A|G|T|C] TG [T|G] C motif previously identified in the promoter region of some *P. penetrans* effectors [14]. This motif was found in the promoter region of 12 genes (Additional file 5: Table S3), representing 40% of the total number of new candidate effectors.

### Presence of orthologues in other plant-parasitic nematodes

A BLAST search conducted with all candidate effectors localized in the gland cells identified here against the draft genome assembly of *P. penetrans* of the same isolate (Vieira and Nemchinov, unpublished), confirmed their nematode origin (0 = e-value < 5.14E-114; Additional file 6: Table S4). Overall, the new candidates were predicted to encode proteins ranging in size from 76 to 806 amino acids (Table 1). All protein sequences were analyzed for the presence of conserved domains using Pfam database searches. Only four candidate effectors were predicted to contain known protein domains: a papain family cysteine protease (PF00112), a pepsin inhibitor-3-like repeated domain (PF06394.1), an astacin (PF01400.21), and a domain of unknown function DUF148 (PF02520.14). For all the remaining candidates no other known functional domains were found. Previously, we have found that several pioneer effectors of *P. penetrans* contained a high number of proline residues. As most of the effectors found here were also novel, we quantified the levels of proline, cysteine and glycine residues. Twelve of the new candidate effectors were rich in proline, while others presented a high content of glycine residues (> 10% of the full protein sequence; Table 1).

**Table 1:** Characterization of candidate gene effector genes specifically localized in the esophageal glands of *Pratylenchus penetrans*. The list is sorted in agreement to their respective fragment per kilobase of transcript per million mapped reads (FPKM) values presented in Additional file 1: Table S1

Transcript code	Interpro Acession	InterPro Name	Pfam	Protein (aa)	Domain position	Domain bit score	E-value	% Cysteine content	% Glycine content	% Proline content
Ppen13114_c0_seq2	–	–	–	87	–	–	–	0.1	16.1	29.9
Ppen8917_c0_seq1	–	–	–	147	–	–	–	0	4.1	10.6
Ppen11800_c0_seq1	–	–	–	86	–	–	–	1.2	10.5	2.3
Ppen9432_c0_seq1	–	–	–	155	–	–	–	3.9	4.5	18.7
Ppen11421_c0_seq1	–	–	–	509	–	–	–	0.2	5.5	4.7
Ppen13553_c0_seq1	–	–	–	116	–	–	–	0.9	23	5.2
Ppen17089_c0_seq1	–	–	–	233	–	–	–	2.6	3.9	2.1
Ppen8388_c0_seq1	–	–	–	94	–	–	–	1.1	19.1	38.3
Ppen12616_c0_seq1	–	–	–	76	–	–	–	11.8	9.2	2.6
Ppen15969_c0_seq2	–	–	–	438	–	–	–	0.7	6.8	3
Ppen13037_c0_seq1	–	–	–	127	–	–	–	0	5.5	4.7
Ppen20090_c0_seq1	–	–	–	128	–	–	–	0	21.1	10.2
Ppen8150_c0_seq1	–	–	–	270	–	–	–	0	9.3	6.3
Ppen11603_c0_seq1	–	–	–	632	–	–	–	0.2	10.1	4.7
Ppen3597_c0_seq1	IPR000668	Papain family cysteine protease	PF00112	375	116–370	153.4	8.60E-45	3.5	9.3	3.2
Ppen16202_c0_seq1	–	–	–	533	–	–	–	0	19.3	22.1
Ppen18231_c0_seq1	–	–	–	388	–	–	–	2.3	6.2	5.4
Ppen10194_c0_seq1	–	–	–	806	–	–	–	0.05	5.5	2.9
Ppen19584_c0_seq1	–	–	–	113	–	–	–	0	23.9	15.9
Ppen14923_c0_seq1	IPR006377	Domain of unknwon function DUF148	PF02520.14	262	48–149	50	2.50E-13	0.1	16.8	5.3
Ppen13972_c0_seq1	–	–	–	141	–	–	–	1.4	7.1	2.8
Ppen16480_c0_seq1	–	–	–	727	–	–	–	0.06	3.2	10.5
Ppen28021_c0_seq1	–	–	–	121	–	–	–	2.5	11.6	16.5
Ppen15256_c0_seq1	IPR010480	Pepsin inhibitor-3-like repeated domain	PF06394.10	248	25–99 | 120–193	58.6 | 78.7	4.6E-16 | 2.5E-22	0.2	6.9	5.2
Ppen16504_c0_seq1	IPR001506	Astacin (Peptidase family M12A)	PF01400.21	564	189–383	156.8	4.20E-46	3	7.4	4.8
Ppen11417_c0_seq1	–	–	–	145	–	–	–	4.8	3.4	1.4
Ppen10830_c0_seq1	–	–	–	173	–	–	–	6.4	5.2	9.8
Ppen18503_c0_seq1	–	–	–	128	–	–	–	1.6	10.9	14.1
Ppen11174_c0_seq2	–	–	–	112	–	–	–	0.9	17.9	3.6
Ppen15571_c0_seq2	–	–	–	405	–	–	–	0.01	5.7	3.2

**Fig. 5:**
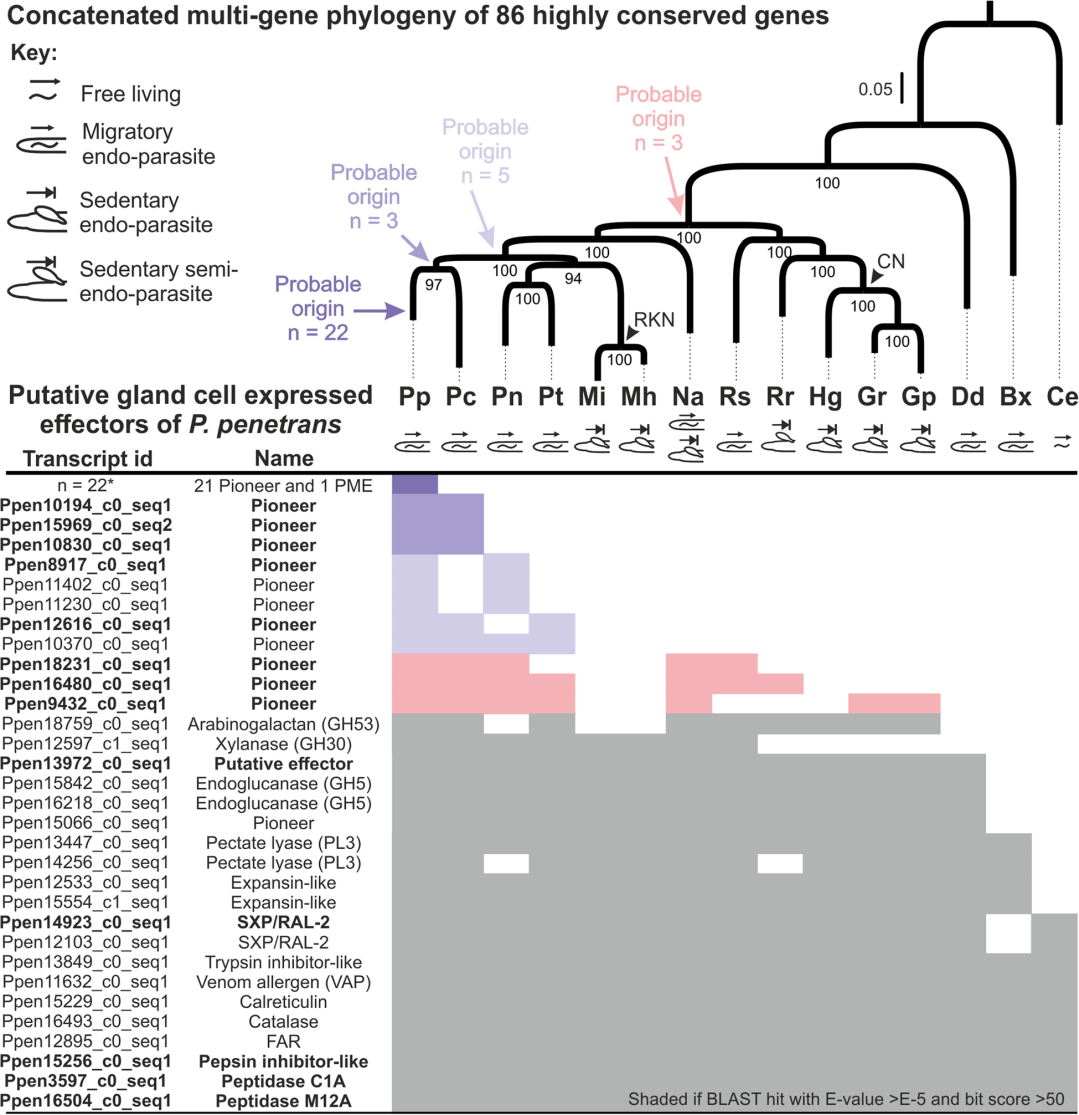
Comparative analyses of the full set of candidate effector genes of *Pratylenchus penetrans* suggest a large-scale effector birth for this species. Top panel corresponds to a schematic phylogeny of the phylum Nematoda based on 86 highly conserved CEGMA genes among plant-parasitic nematodes with different parasitism strategies and the free-living nematode *Caenorhabditis elegans* (Ce). Shaded squares in the lower panel indicate significant blast hits of each nematode species against the set of effectors identified for *P. penetrans.* The new candidate effectors identified in this study are represented in bold (*n* = 13) while the remaining 17 genes are represented within the set of 22 effectors found specifically for *P. penetrans* (marked with an asterisk)*.* n, represents the putative number of gene births in each nematode speciation cluster. Pp: *Pratylenchus penetrans;* Pc: *Pratylenchus coffeae*; Pn: *Pratylenchus neglectus*; Pt: *Pratylenchus thornei*; Mi: *Meloidogyne incognita*; Mh: *Meloidogyne hapla*; Na: *Nacobbus aberrans*; Rs: *Radopholus similis*; Rr: *Rotylenchulus reniformis*; Hg: *Heterodera glycines*; Gr: *Globodera rostochiensis*; Gp: *Globodera pallida*; Dd: *Ditylenchus destructor*; Bx: *Bursaphelenchus xylophilus;* Ce: *Caenorhabditis elegans.* RKN: root-knot nematodes; CN: cyst nematodes

The identification of a more complete roster of candidate effectors in *P. penetrans,* coupled with available genome and transcriptome sequences for many phylogenetically well-positioned species, provides an opportunity to investigate the evolutionary history of the effector repertoire. We reconstructed a robust multi-gene phylogenetic tree, based on 86 CEGMA (Core Eukaryotic Genes Mapping Approach) genes highly conserved in the following species: root lesion nematodes *Pratylenchus coffeae* [23–25], *P. neglectus,* and *P. thornei* (PRJNA512537 [26];), and the burrowing nematode *Radopholus similis* [27]; root-knot nematodes *Meloidogyne incognita* [28] and *M. hapla* [29]; cyst nematodes *Globodera pallida* [30], *G. rostochiensis* [11], and *Heterodera glycines* [31]; the false root-knot nematode *Nacobbus aberrans* [32], the reniform nematode *Rotylenchulus reniformis* [33], the migratory potato rot nematode *Ditylenchus destructor* [34]; the pinewood nematode *Bursaphelenchus xylophilus* [35]; and the free-living nematode *Caenorhabditis elegans* (http://parasite.wormbase.org). We then searched for the presence of putative homologs of the entire *P. penetrans* putative effector repertoire validated by in situ hybridization (*n* = 53) in all these species using BLAST (e-values < 1 × 10^− 5^ and coverage > 50, Fig. 5).

Two noteworthy trends were apparent: 1) large scale effector birth in the Pratylenchidae in general and *P. penetrans* in particular, and 2) large scale effector death in root-knot nematodes (in some cases convergent loss with cyst nematodes). As evidence of the former, and despite the inclusion of three other *Pratylenchus* species in the analysis and the application of a relatively relaxed similarity threshold, 41% of the putative effectors (and 65% of the pioneer effectors) were unique to *P. penetrans* (c.f. 0.22% of all transcripts and 0.29% of all pioneer transcripts). The most parsimonious explanation for this observation is a dramatic de novo effector gene birth (i.e. not by neofunctionalization) since the divergence from the last common ancestor in the phylogeny. Only three putative effectors were common to both *P. penetrans* and its three most closely related Pratylenchidae species included in the analysis (*P*. *coffeae*, *P. neglectus* and *P. thornei*). Five putative effectors were common to three of the four *Pratylenchus* species, possibly indicative of disparate gene loss in the Pratylenchidae and in root-knot nematodes (given the monophyletic relationship with Pratylenchidae). Notably, two pioneer effectors were generally common to root lesion nematodes, the false root-knot nematode, the burrowing nematode, and the reniform nematode, but absent in sedentary endoparasitic nematodes, such as root-knot and cyst nematodes. The simplest possible explanation could be an ancient origin of the gene, in the last common ancestor of these species and subsequent convergent loss in sedentary endoparasitic root-knot and cyst nematodes. A final pioneer gene appears to have a similarly ancient origin with disparate loss. Several other putative effectors have homologues distributed across the tree, many of which have known annotations, and together likely represent evidence for neofunctionalization (when not putatively originated from horizontal gene transference events as, for example, different families of CWDEs).

### Expression of selected genes during nematode-plant interaction

**Fig. 6:**
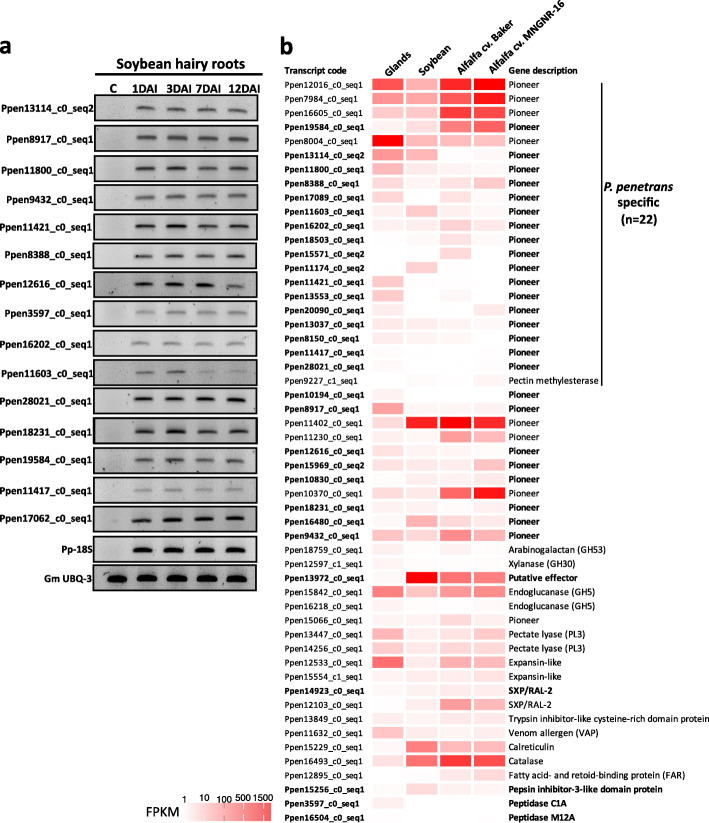
Expression profile of *Pratylenchus penetrans* candidate effector genes in *planta*. **a** Semi-quantitative reverse transcription PCR validating the expression levels of 15 gland-cell localized nematode genes on soybean hairy roots at 1, 3, 7 and 12 days after nematode infection. As a positive control, all cDNA libraries were amplified with primers derived from the *18S* gene of *P*. *penetrans* or the *Ubiquitin-3* (UBQ-3) gene of *Glycines max.* C corresponds to non-infected hairy roots. **b** Heat maps representing the expression profile of the full set of 53 candidate effectors identified so far for *P. penetrans* using the fragment per kilobase of transcript per million mapped reads (FPKM) values of the current gland-cell library (Glands), and public messenger RNA-sequencing datasets originating from total RNA extracted from soybean (BioProject ID PRJNA304159 [7]) and alfalfa plants (cultivars Baker and MNGNR-16; BioProject ID PRJNA547347 [13]) infected with the same *P. penetrans* isolate at 7 DAI. The new candidate effectors identified in this study are represented in bold

To validate the results of in silico analysis, we examined the expression of 15 genes in soybean hairy roots at 1, 3, 7 and 12 days after nematode inoculation (DAI). All of the selected genes could be detected within the nematode-infected roots and their expression varied (Fig. 6a) in accordance with the patterns observed during in silico analysis. To get a broader insight into the expression of all candidate effectors validated so far in *P. penetrans*, we took advantage of the transcriptome data previously generated from different plants, i.e., two different cultivars of alfalfa and soybean, infected with the same isolate (Fig. 6b). Heat maps that were used to visualize the expression profile confirmed the transcription of a large set of effectors during nematode interactions with the roots. A core set of effectors was prominently induced (FPKM > 100) during infection independently of the host genotype (e.g., a catalase, an expansin-like gene, an endoglucanase, and several pioneer genes). These results suggested that a complex network of effectors is actively transcribed in the course of infection.

## Discussion

In this work, we conducted a comprehensive screening of potential effector proteins of the agriculturally important nematode species *P. penetrans*. This was accomplished using a tissue-specific transcriptome sequencing approach to identify transcripts abundantly expressed in the esophageal gland cells and encoding proteins likely secreted during nematode parasitism. A substantial number of reads were generated and an extensive catalogue of transcripts specific to the gland cells compiled. Comparative analyses of the gland cell transcriptome confirmed most of the previously described cases of gland-cell expression and, most importantly, drastically expanded the size of the roster of gland-cell expressed genes to double its previous value. Importantly, this is still likely an underestimate for *P. penetrans* due to the stringent cut-off used: other candidate effectors with lower levels of expression and established roles in parasitism were also present in this library.

Of the new candidates identified, only four genes had a predicted functional domain or known annotation. Among them, transcripts encoding a second member of the SXP/RAL-2 family were validated to be gland-specific. Members of this gene family are characterized by the presence of the DUF148 protein domain and can be found across different clades of nematodes [36]. In PPNs, SXP/RAL-2 transcripts showed distinct localization in the subventral esophageal glands of the root-knot nematode *M. incognita* [37] or in the epidermis and amphidial sheet cells of the cyst nematode *G. rostochiensis* [38]. The biological functions of the different members of this family are still lacking. However, their high abundance in the glands and active transcription during plant infection provides additional insight regarding their significance during nematode parasitism. In addition, two proteases and one protease inhibitor-like protein were validated specifically in the glands: M12 astacin and a papain cysteine protease and pepsin inhibitor-3-like domain-containing protein, respectively. To date, several studies have evidenced the role of different classes of proteases in other plant-pathogenic interactions [39]. In *M. incognita*, expression of cysteine and aspartyl proteases, potentially involved in softening of plant cell walls during migration, pre-digestion of nutrients or inactivation of plant defense proteins, was confirmed in the esophageal glands [40, 41]. In animal-parasitic nematodes, different classes of proteases and protease inhibitors have been identified among the main components of excretory/secretory products of different species [42]. For example, M12 astacins, which are involved in skin penetration and migration [43], are widely distributed in nematodes belonging to the clade IV. The secretion of these proteases has been linked to diverse functions, including host tissue penetration, modification of the host environment, destruction of plant defense proteins and digestion [43]. Protease inhibitors on the other hand may be involved in the protection against degradation by host proteases or manipulation of the host responses. The identification of new gland-specific proteases and protease inhibitors in *P. penetrans*, and other PPNs, supports the idea that these proteins may have an active role during plant parasitism.

The lack of recognizable Pfam domains is a hallmark of PPN effectors. The fact that a large proportion of transcripts found in this work belongs to novel genes (> 80%) highlights the uniqueness of *P. penetrans*. To advance our understanding of these pioneer effectors of *P. penetrans*, we adopted a phylogenetic approach and compared the expanded effector repertoire of *P. penetrans* to the genome/transcriptome of other nematode species with similar or contrasting parasitism strategies. With the caveat that the absence of evidence is not always evidence of absence, the consistency of the patterns of presence and absence allowed us to inferred plausible evolutionary histories that shaped the effector repertoires of not just *P. penetrans*, but also other species in the phylogeny. For example, the dominant majority of pioneer effectors were restricted to *P. penetrans*, thus, indicating substantial de novo gene gain (i.e., not neofunctionalization of the existing genetic pool). Whether this dramatic gene gain is linked to the broad host range of this species remains to be determined. At the opposite end of the spectrum, many non-pioneer effectors (i.e. those with recognizable domains) have homologues across the phylogeny, in some cases even in non-parasitic species. In these cases, it is likely that neofunctionalization has occurred to give rise to new effector genes in *P. penetrans*. However, with the currently available information does not yet allow these events to be dated. The instances between gene gain and functional diversification are similarly informative. Given the monophyly of root-knot nematodes (Meloidogynidae) with Pratylenchidae, effector genes that are generally conserved across the Pratylenchidae but absent in root-knot nematodes, can be indicative of secondary loss. These cases (five discovered in this study) are part of a larger trend of effector gene loss in root-knot nematodes, including those originated before the split from cyst nematodes (Heteroderidae). One particularly interesting example (*Ppen16480_c0_seq1*) appears to be a pioneer gene that is present only in species with migratory lifestyles (root lesion, burrowing and the false root-knot nematodes). Given its wide distribution among these species, the most parsimonious explanation could be an ancient origin and convergent loss in sedentary endoparasites. Although some effector candidates, for instance those similar to the arabinogalactan (GH53), have comparable distributions in other PPNs, the same conclusions can unlikely be drawn because their spread could be equally well explained by multiple horizontal transfer events.

The stringent cut-off criteria used in this work while enriching for likely secreted candidate effectors, could also provide a hint on transcripts specific to the gland cell machinery (e.g. chaperones responsible for packaging effector proteins). Therefore, transcripts encoding proteins without a canonical signal peptide should not be ignored. Moreover, there is evidence that some effectors lacking a conventional signal peptide, for instance: Mi-14-3-3 [44], Mi-GST-1 [45] and MiPFN3 [46], play important roles in parasitism of the root-knot nematode *M. incognita*. The cyst nematode effector HsIPT, which also lacks a signal peptide, is involved in the activation of the host cell cycle of the syncytium cells [47]. Transcripts encoding proteins without a canonical signal peptide, such as ShK domain-like proteins, were found in this work to be highly abundant and localized specifically in the nematode esophageal glands. ShK-domain containing proteins secreted by animal-parasitic nematodes have a role in immuno-modulatory activity [48]. Although the mechanisms of protein secretion into the plant cell in the absence of a genuine signal peptide are still unknown, it cannot be ruled out, at least for *P. penetrans*, that some of these proteins may be part of the molecular machinery of the esophageal glands rather than effectors per se. However, effector proteins of PPNs are often identified in the secretory granules within the esophageal glands [49, 50]. It remains unknown if the contents of such vesicles can be directly released into the host cells as a complex network of proteins. Validation of secretion of such types of proteins by nematodes into the host using, for example, effector-specific antibodies [41, 51, 52], will provide crucial information regarding their molecular involvement with the host.

Since *P. penetrans* is one of the most successful species in the genus and is able to infect a large number of plants, high diversity of its effectors may potentially create a strong molecular basis for this broad host range. One of the common features of host infection by RLNs is the massive damage induced in the root cortex as a consequence of migration and feeding activity by these nematodes. Despite a severe impact on root tissues, plants seem to be unable to mount strong defenses (e.g., cell death) to block the progression of nematodes [7]. The diversity of effectors highly abundant in the glands is likely to be related to the wide range of molecular functions that are required for penetration and invasion of host roots, detoxification, suppression of host defenses and many other unidentified functions. The RNA-seq libraries previously generated from nematode-infected roots served to support differential gene expression of all candidate effectors validated so far for *P. penetrans*. Plant signals may be required to trigger specific nematode responses, including secretion of effector proteins. For example, regulation of different CWDE genes of *P. coffeae* is host-specific [25]. Host-dependent response could also explain differential expression profiles observed for the set of *P. penetrans* effectors. Nevertheless, there is a possibility that these differences may result from the limited number of nematode reads recovered from nematode-infected roots. Since *P. penetrans* is a polyphagous species, comparative analyses of gland transcriptomes originated from nematodes parasitizing on different host plants could support the dynamics of effector expression relative to the host-specific responses. It may be that to define a truly comprehensive repertoire of effectors for a polyphagous nematode species, like *P. penetrans*, would require gland cell sequencing of nematodes collected from a variety of hosts.

## Conclusions

Altogether, our data demonstrate that *P. penetrans* deploys a novel repertoire of effectors during its interaction with the host. Notable, 26 out of the 30 (86%) new candidate effectors identified in this study represent genes without known domains, which demonstrates the lack of knowledge about this group of nematodes and warrants further investigation. Future efforts should focus on the identification and characterization of the host targets of these effectors to determine their biological roles and provide crucial information for application of genetic engineering strategies for the control of PPNs. Furthermore, the results reported in this study may also contribute towards basic understanding of the adaptation of *P. penetrans*, and other RLNs, to specific host plants.

## Methods

### Nematode isolate

*Pratylenchus penetrans* isolate (NL 10p RH) collected in Beltsville (Maryland, U.S.) was maintained under sterile conditions in soybean hairy roots (*Glycine max*) growing on agar plates with Murashige and Skoog (MS) medium. Nematodes were transferred every 2 months to new soybean hairy roots and kept in the dark at 25 °C.

### Gland-cell cDNA library of *Pratylenchus penetrans*

Mixed nematode life stages were collected using the Baermann funnel technique from hairy roots in vitro stocks and washed in PBS buffer. *Pratylenchus penetrans* gland cells were collected from a pool of 100 mixed dorsal and subventral gland cells as previously described [10]. Gland cells RNA was extracted using the Arcturus PicoPure RNA isolation kit (Arcturus Bioscience). Total RNA (≤ 1 ng) was used directly as input for the SMARTer Stranded Total RNA-Seq kit - Pico Input Mammalian (Clontech). The *P. penetrans* RNA-seq library was then submitted for sequencing to Admera Health (South Plainsfield, NJ) and sequenced by Illumina NextSeq 500. The paired-end library totaled 147,906,800 raw reads and the quality of the library was assessed with the program FASTQC (https://www.bioinformatics.babraham.ac.uk/projects/fastqc/). The vast majority of the reads were greater than 144 bp in length. The GC content averaged 47%. The median quality value ranged from 32 to 40 over the length of the entire read with an overall per average sequence quality of 40. The reads were also free of adapter content.

### Gene expression analyses

Illumina RNA-seq reads of the esophageal gland cells were initially trimmed and mapped to the 23,715 transcripts generated for the same *P. penetrans* isolate [13] using CLC Genomics v. 8 with default parameters. Gene expression patterns were deduced from the aligned reads and determined as Fragments Per Kilobase of Transcript per Million mapped reads (FPKM) values. The expression data were partitioned into expression bins and enrichment in secreted proteins for each expression bins was assessed using a hypergeometric test with the phyp-function available in R (v3.5.2). The FDR (False Discovery Rate) was calculated according to [53] with the *p*-adjust function available in R. The expression bins were considered as significantly enriched in secreted protein when FDR < 0.001.

BLASTp searches were carried out against the NR database at NCBI (e-value cutoff of 1e^− 5^ and bitscore > 50). Interpro were performed for the predicted proteins of the set of 230 transcripts using BLAST2GO [54] with default parameters. PFAM domain searches were performed using the Pfam dataset Pfam-A v32 obtained at https://pfam.xfam.org [55] and run through CLC Main Workbench v.7. SIGNALP v. 4.0 was used to confirm the presence/absence of protein signal peptide in the genome of the predicted proteins [56], and transmembrane domains were predicted using TMHMM server version 2.0 (http://www.cbs.dtu.dk/services/TMHMM/). Cysteine, glycine and proline contents were calculated for each predicted mature protein with CLC Main Workbench v.7.

### In situ hybridization assays

Total RNA was extracted from a pool of mixed stages of *P. penetrans* using the RNeasy Plant Mini kit (QIAGEN) according to the manufacturer’s instructions. RNA was treated with RNase-free DNase (QIAGEN) before reverse transcription. The quantity and quality of the extracted RNA was assessed by a ND-1000 NanoDrop spectrophotometer (Thermo Scientific) and cDNA was synthesized using the iScript first-strand synthesis kit (Bio-Rad) following the manufacturer’s instructions. Whole mount in situ hybridizations were performed in all stages of *P. penetrans* following the protocol of [57]. Specific primers were designed to amplify a range of gene products (104 to 305 bp) using the cDNA library produced from the mix pool of *P. penetrans* stages (Additional file 7: Table S5). The resulting PCR products were then used as a template for generation of sense and antisense DIG-labeled probes using a DIG-nucleotide labeling kit (Roche). Hybridized probes within the nematode tissues were detected using an anti-DIG antibody conjugated to alkaline phosphatase and its substrate. Nematode segments were observed using a Nikon Eclipse 5*i* light microscope.

### Promoter analyses

To identify the non-coding promoter motif previously determined for several effector of *P. penetrans* [14], approximately 600 nucleotides of the 5′ sequence from the start codon were manually extracted based on BLASTn coordinates against the draft genome of the same nematode isolate (Vieira and Nemchinov, unpublished). These promoter regions were screened for the presence of the motif consensus CAA [A|G|T|C] TG [T|G]C.

### BLAST hit analyses against other nematode genomes/transcriptomes

Focusing on a subset of candidate effectors with verified esophageal gland-cell expression in *P. penetrans*, additional in silico analyses were performed. Open reading frames were used to perform BLASTn searches (e-value >1e^− 10^) against the draft genome of the same *P. penetrans* isolate (Vieira and Nemchinov, unpublished). The top hit sequences were manually examined and each transcript sequence was aligned to the respective genomic scaffold using MUSCLE [58]. Genomic and transcript sequences were submitted to FGENESH (www.softberry.com) for gene and protein prediction using *Caenorhabditis elegans* as model [59].

Transcripts highly represented in the gland cell library were additionally compared using BLASTx and tBLASTx (e-value cutoff of 1e^− 5^ and bitscore > 50) to a set of transcriptomes and genomes of PPNs publicly available at NCBI and Wormbase (http://parasite.wormbase.org). This set comprised sequenced genomes of PPNs from different clades [60]: 1) Clade 12B: sedentary species such as root-knot nematodes *Meloidogyne incognita* [28] and *M. hapla* [29]; cyst nematodes *Globodera pallida* [30], *G. rostochiensis* [11], and *Heterodera glycines* [31]; the false root-knot nematode *Nacobbus aberrans* [32], the reniform nematode *Rotylenchulus reniformis* [33], and the draft genome of the burrowing nematode *Radopholus similis* [27]; 2) Clade 12A: the migratory potato rot nematode *Ditylenchus destructor* [34]; 3) Clade 10: the pinewood nematode *Bursaphelenchus xylophilus* [35]; and 4) Clade 9A: the free-living nematode *C. elegans* (http://parasite.wormbase.org). Local tBLASTn searches were performed against the transcriptomes of additional Pratylenchidae species (Clade 12B), such as *P. coffeae* [23–25], *P. neglectus* and *P. thornei*. For the last two species de novo assemblies were generated using the corresponding raw reads deposited at NCBI Sequence Read Archive (SRA) BioProject PRJNA512537 [26]. Eighty-six CEGMA genes conserved in the genome and or transcriptome resources of all 15 nematodes species described above were used for phylogenetic reconstruction (Additional file 7: Table S6). Protein sequences of individual CEGMA genes were aligned and refined using MUSCLE [58]. Alignments were concatenated and model selection for each partition was carried out using the IQtree server. A concatenated multi-gene phylogeny was generated using the ultra-fast mode and 1000 bootstraps [61].

### Differential expression of *Pratylenchus penetrans* candidate effectors *in planta*

Semi-quantitative RT-PCR analyses were performed using total RNA extracted from nematode-infected soybean hairy roots at different time points following the same methodology as described by [15]. The same set of primers selected for in situ hybridization were used for nematode transcript amplification, while the following genes were used as references: *Ubiquitin-3* (Ubi3) for soybean hairy roots and *18S* for *P. penetrans* (Additional file 7: Table S5). The abundance of the full set of candidate effectors were estimated as FPKM values and presented as heatmaps using previously generated transcriptome data for *P. penetrans-*infected plants, i.e. soybean (SRA BioProject PRJNA304159 [13]) and two alfalfa (*Medicago sativa* L.) cultivars (Baker and MNGNR-16, SRA BioProject PRJNA547347 [7]).

## Supplementary information

Additional file 1: Table S1. Summary of BLAST hit analyses of the top 230 most abundant transcripts of *Pratylenchus penetrans* encoding putative secreted proteins (FPKM > 8) originated from the esophageal gland cells library. BLAST analyses were performed against the non-redundant GenBank database. Transcript abundance was determined as Fragments Per Kilobase of Transcript per Million mapped reads (FPKM) values. (XLSX 30 kb)

Additional file 2: Table S2. List of transcripts encoding putative effector proteins with FPKM values below the established cut-off (FPKM < 8) originated from the esophageal gland cells library. (XLSX 14 kb)

Additional file 3: Fig. S1. In situ hybridization assays using the corresponding sense DIG-labeled probes (control) for 30 candidate effector genes detected within the esophageal glands of *Pratylenchus penetrans.* Details regarding each gene annotation and description are presented in the same order as sorted in Table 1. Bars = 20 μm. (TIFF 5250 kb)

Additional file 4: Fig. S2. In situ hybridization assays using the corresponding sense DIG-labeled probes (control) for transcripts encoding: (**a**) ShK domain-like protein, and (**b**) a 14–3-3 protein. Bars = 20 μm. (TIFF 656 kb)

Additional file 5: Table S3. List of promoter sequences extracted for 30 genes with gland cell localization in *Pratylenchus penetrans.* The 600 nt of the upstream region of the start codon was queried for the presence of the CAA[AG|T|C]TG[T|G] C motif previously found associated with gland cell expression of other candidate effectors of *P. penetrans. (XLSX 17 kb)*

Additional file 6: Table S4. BLAST hit analyses of transcripts with gland cell localization against the draft genome of *Pratylenchus penetrans. (XLSX 11 kb)*

Additional file 7: Table S5. List of primers used in this study. (XLSX 14 kb)

Additional file 8: Table S6. List of CEGMA genes used in this study. (XLSX 693 kb)

## Data Availability

Raw RNAseq reads generated in this publication are available at National Center for Biotechnology Information (NCBI), under the SRA accession number PRJNA668165.

## Abbreviations

CWDEs: cell wall-degrading enzymes
DAI: Days after inoculation
FPKM: Fragments per Kilobase per Million Mapped Reads
NR: Non-redundant database
nt: nucleotide
PPN: Plant-parasitic nematodes
RLN: root lesion nematodes
RNA-seq: Sequencing of RNA
